# Effects of the Non-Nutritive Sweeteners on Glucose Metabolism and Appetite Regulating Hormones: Systematic Review of Observational Prospective Studies and Clinical Trials

**DOI:** 10.1371/journal.pone.0161264

**Published:** 2016-08-18

**Authors:** Alonso Romo-Romo, Carlos A. Aguilar-Salinas, Griselda X. Brito-Córdova, Rita A. Gómez Díaz, David Vilchis Valentín, Paloma Almeda-Valdes

**Affiliations:** 1 Instituto Nacional de Ciencias Médicas y Nutrición Salvador Zubirán, Department of Endocrinology and Metabolism, México City, México; 2 Medical Research Unit in Clinical Epidemiology, UMAE Hospital de Especialidades, Centro Médico Nacional Siglo XXI, Instituto Mexicano del Seguro Social (IMSS), México City, México; University of Lancaster, UNITED KINGDOM

## Abstract

**Background:**

The effects of non-nutritive sweeteners (NNS) on glucose metabolism and appetite regulating hormones are not clear. There is an ongoing debate concerning NNS use and deleterious changes in metabolism.

**Objectives:**

The aim of this review is to analyze the scientific available evidence regarding the effects of NNS on glucose metabolism and appetite regulating hormones.

**Data Sources and Study Eligibility Criteria:**

We identified human observational studies evaluating the relation between NNS consumption and obesity, diabetes, and metabolic syndrome, in addition to clinical trials evaluating the effects of NNS in glucose metabolism and appetite regulating hormones.

**Results:**

Fourteen observational studies evaluating the association between NNS consumption and the development of metabolic diseases and twenty-eight clinical trials studying the effects of NNS on metabolism were included. Finally, two meta-analyses evaluating the association between the consumption of NNS-containing beverages and the development of type 2 diabetes were identified.

**Conclusions:**

Some observational studies suggest an association between NNS consumption and development of metabolic diseases; however, adiposity is a confounder frequently found in observational studies. The effects of the NNS on glucose metabolism are not clear. The results of the identified clinical trials are contradictory and are not comparable because of the major existing differences between them. Studies evaluating specific NNS, with an adequate sample size, including a homogeneous study group, identifying significant comorbidities, with an appropriate control group, with an appropriate exposure time, and considering adjustment for confounder variables such as adiposity are needed.

## Introduction

The prevalence of obesity has more than doubled since 1980; in parallel in 2014, the estimated number of patients with diabetes in the world was 385 million with a projection to increase to 592 million by 2035. One of the contributing factors attributed to the increase in obesity, type 2 diabetes and other metabolic diseases is the consumption of a high sugar/high fat diet [1]. To avoid the negative health conditions associated with the excessive sugar intake, there has been an upsurge in the consumption of nonnutritive sweeteners (NNS) as an alternative [2]. At this time six NNS, sucralose, aspartame, saccharin, acesulfame-K, neotame, and advantame, are approved to be used as sweeteners in food, and two naturally derived NNS, steviol glycosides and Luo han guo extract, are generally recognized as safe and endorsed for use in food by the US Food Drug Administration (FDA) and the European Food Safety Authority (EFSA) [3, 4]. Nowadays, they are globally used and they are found in several products.

Recently, the EFSA conducted a re-evaluation of aspartame safety, and concluded that aspartame and its breakdown products are safe for the general population (including infants, children and pregnant women) [4]. Before the FDA approved NNS consumption, a series of toxicological and clinical studies in a number of species, including humans, were conducted to demonstrate that they are generally safe and well-tolerated [5]. There is an ongoing debate over whether NNS use may be associated to deleterious metabolic changes in humans [6]. This article aims to collect the information regarding the effects of NNS consumption on metabolic diseases, based on a systematic review of the scientific literature.

## Study Search and Selection

We identified human studies evaluating the effects of NNS consumption in metabolic conditions through systematic searches and hand searches on April 8, 2015 (updated on March 25, 2016) in three electronic databases: PubMed, The Cochrane Library, and Trip Database. We conducted the search for observational studies to answer the following research question: Is there a relation between NNS consumption and the development of metabolic chronic diseases in adults? For clinical trials, we directed the search to answer the next research question: Is there an effect of NNS on glucose metabolism and appetite regulating hormones compared to water or other sweeteners in adults? The terms used in the systematic search were those related to NNS and artificially sweetened beverages including the next Medical Subject Headings (MeSH) terms: artificial sweeteners / non-nutritive sweeteners / carbonated beverages / sucralose / aspartame / stevia / saccharin / acesulfame potassium / diet soda / diabetes mellitus / obesity / metabolic syndrome. To complement the search, we also performed a hand-searching strategy through certain journals and references in other articles. Time and language of publication were not restricted. Inclusion criteria consisted in original studies of prospective design conducted in adult humans. For cohort studies, we considered those that evaluate the association between NNS consumption and the development of diabetes, metabolic syndrome or obesity, with a follow up of at least three years. For clinical trials we included those that evaluate the effects of any NNS on outcomes related to glucose metabolism and appetite regulating hormones (S1 File). One researcher (AR) screened the articles titles and abstracts to remove those that easily were detected to be not related to the objective of this review, and three researchers (AR, PA, and GB) read the articles that could be eligible in the systematic review and select those that finally are included. Articles evaluating the effects of NNS in other conditions or evaluating other outcomes not related were excluded.

## Results

### Literature search

**Fig 1:**
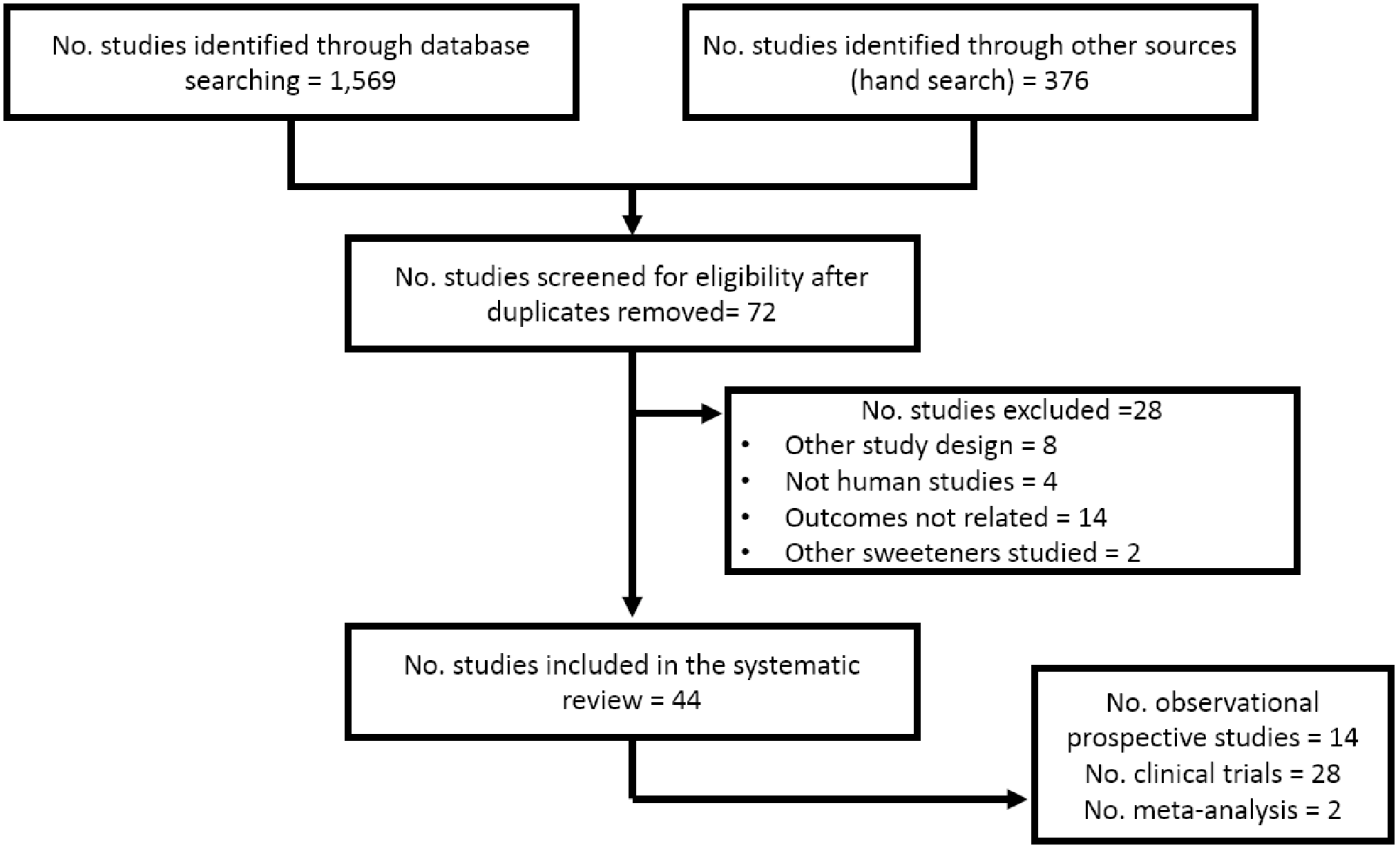
Flow diagram of the systematic search.

We identify 1569 studies through database searching; in addition, 376 were found by the hand searching strategy. After duplicates removal and initial screening, 72 studies were reviewed. Finally, 44 studies were included after the exclusion of 28 that did not fulfill the inclusion criteria. Fig 1 shows the flow chart describing the process of the systematic search.

### Observational studies

We included fourteen observational studies evaluating the association between NNS consumption and the development of metabolic diseases including type 2 diabetes, obesity, and metabolic syndrome. All of these studies have considered NNS consumption in beverages and most of them in soft drinks.

Summarizing the results, the majority of these studies have found significant associations between the ingestion of NNS and the development of metabolic diseases. Among these studies there are two reports derived from the Nurses’ Health Study (NHS I and II) that included more than 70,000 and 90,000 women, with an average follow-up of 24 and 8 years for the first and the second studies, respectively. The first of these studies found a significant association between caffeinated artificially sweetened beverages consumption and development of type 2 diabetes (RR 1.35, 95% CI 1.24–1.47). However, this association was lost after the adjustment for body mass index (BMI) and energy intake (RR 1.01, 95% CI 0.93–1.10) [7]. In the NHS II no association was found [8].

Another large cohort study that evaluated the effect of artificially sweetened beverages consumption and the development of type 2 diabetes is the Health Professionals Follow-Up Study. This included approximately 40,000 male health professionals followed over 20 years. This study found a significant association between NNS consumption and type 2 diabetes development, even after multivariable adjustment (HR 1.40, 95% CI 1.26–1.56). However, this association was lost after the adjustment for BMI (HR 1.09, 95% CI 0.98–1.21) [9].

The European Prospective Investigation into Cancer and Nutrition (EPIC) Study, performed in eight European countries, included 340,234 men and women. This study reported a significant association between artificially sweetened soft drinks ingestion and type 2 diabetes development (HR 1.93, 95% CI 1.47–2.54). This association was attenuated after multivariable adjustment (HR 1.88, 95% CI 1.44–2.45), and lost statistical significance after further adjustment for BMI and energy intake (HR 1.13, 95% CI 0.85–1.52) [10].

Table 1 shows a summary of the results of the included cohort studies. On Table 2 the crude and adjusted risks reported in these studies are contrasted.

**Table 1:** Observational studies evaluating the association between artificially sweetened beverages consumption and the risk for development of metabolic diseases.

Author, year, cohort, and country	Follow-up time	Population (Number and age)	Results
Schulze MB, et al. 2004 The Nurses’ Health Study (NHS II) USA [8]	8 years	91,249 women 24–44 years	741 incident cases of T2D No significant association between the consumption of one or more diet soft drinks per day and development of T2D after adjustment for baseline BMI (RR: 1.21; 95% CI: 0.97–1.50; P = 0.12)
Dhingra R, et al. 2007 The Framingham Heart Study USA [11]	4 years	6,039 adults Mean age 52.9 years	1,239 incident cases of metabolic syndrome Association between the consumption of one or more diet soft drinks per day and the development of metabolic syndrome after multivariable adjustment (OR: 1.53; 95% CI: 1.10–2.15) compared with the consumption of less than one soft drink per week. However, the adjustment did not include BMI or waist circumference
Lutsey PL, et al. 2008 The Atherosclerosis Risk in Communities (ARIC) Study USA [12]	9 years	9,514 adults 45–64 years	3,782 incident cases of metabolic syndrome Consumption of artificially sweetened beverages (third tertile) associated with incident metabolic syndrome after multivariable adjustment without consideration of adiposity (HR: 1.34; 95% CI: 1.24–1.44; P<0.001)
Fowler SP, et al. 2008 The San Antonio Heart Study (SAHS) USA [13]	7–8 years	3,682 adults 25–64 years	1,250 incident cases of overweight or obesity (BMI ≥25 kg/m^2^) in people with normal weight at the beginning of the study (BMI <25 kg/m^2^) Significant association between the consumption of artificially sweetened beverages (fourth quartile) and the incidence of obesity (OR: 2.03; 95% CI: 1.36–3.03; P<0.001) Positive dose-response relationship with the changes in BMI during the study (OR: 1.78; 95% CI: 1.51–2.06; P<0.0001)
Palmer JR, et al. 2008 The Blacks Women’s Health Study (BWHS) USA [14]	4 years	43,960 women 21–69 years	906 incident cases of T2D No relationship between the consumption of one or more diet soft drinks per day and the risk of T2D (RR: 1.06; 95% CI: 0.83–1.36) compared with the consumption of less than one diet soft drink per month
Nettleton JA, et al. 2009 The Multi-Ethnic Study of Atherosclerosis (MESA) USA [15]	7 years	5,011 adults 45–84 years	871 incident cases of metabolic syndrome and 413 of T2D Intake of one or more servings per day of diet soda was positively associated with incident metabolic syndrome (HR: 1.36; 95% CI: 1.11–1.66; P<0.001). However, after adjustment by adiposity (BMI and waist circumference) the association was not significant (HR: 1.17; 95 CI: 0.96–1.44; P = 0.06) Intake of one or more servings per day of diet soda was positively associated with incident T2D (HR: 1.67; 95% CI: 1.27–2.20; P<0.001). However, after adjustment for adiposity (BMI and waist circumference) the association was attenuated remaining statistically significant (HR: 1.38; 95 CI: 1.04–1.82; P = 0.01)
de Koning L, et al. 2011 The Health Professionals Follow-Up Study (HPFS) USA [9]	20 years	40,389 men 40–75 years	2,680 incident cases of T2D Association of artificially sweetened beverages consumption (fourth quartile, median consumption of 6.5 servings per week) and the development of T2D (HR: 1.91; 95% CI: 1.72–2.11; P<0.01). However, in the multivariate model the association was not conserved (HR: 1.09; 95% CI: 0.98–1.21; P = 0.13)
Duffey KJ, et al. 2012 The Coronary Artery Risk Development in Young Adults (CARDIA) Study USA [16]	20 years	3,728 adults 18–30 years	The non-consumers of diet beverages had lower risk for developing metabolic syndrome compared to consumers (HR: 0.81; 95% CI: 0.69–0.95; P<0.05)
Bhupathiraju SN, et. al. 2013 The Nurses’ Health Study I (NHS I) USA [7]	24 years	74,749women 30–55 years	7,370 incident cases of T2D No association between the consumption of one or more servings per day of caffeinated artificially sweetened beverages and the development of T2D after multivariable adjustment, including BMI and energy intake (RR: 1.01; 95% CI: 0.93–1.10; P = 0.99) The consumption of caffeine-free artificially sweetened beverages was associated with higher risk of T2D after multivariable adjustment for BMI and energy intake (RR: 1.09; 95%CI: 1.00–1.18; P = 0.02)
Bhupathiraju SN, et. al. 2013 The Health Professionals Follow-Up Study (HPFS) USA [7]	22 years	39,059 men 40–75 years	2,865 incident cases of T2D No association between the consumption of one or more servings per day of caffeinated artificially sweetened beverages and the development of T2D after multivariable adjustment including BMI and energy intake (RR: 1.06; 95% CI: 0.93–1.22; P = 0.55); and also for caffeine-free artificially sweetened beverages (RR: 1.15; 95% CI: 0.99–1.33; P = 0.06)
The InterAct Consortium 2013 The European Prospective Investigation into Cancer and Nutrition (EPIC) Study Eight European countries [10]	16 years	340,234 adults 39–69 years	11,684 incident cases of T2D Significant association between the consumption of one or more servings per day of artificially sweetened soft drinks and the development of T2D (HR: 1.84; 95% CI: 1.52–2.23; P<0.0001). However, after multivariable adjustment including BMI and energy intake, the association did not remained statistically significant (HR: 1.13; 95% CI: 0.85–1.52; P = 0.24)
Fagherazzi G, et al. 2013 Epidemiologic study of French female teachers (E3N) in the EPIC Study France [17]	14 years	66,118 women Mean age 52.6 years	1,369 incident cases of T2D Consumption of more than 603 mL per week of artificially sweetened beverages associated with incident T2D after multivariable adjustment including BMI (HR: 1.68; 95% CI: 1.19–2.39; P = 0.0057)
Sakurai M, et al. 2014 Employee health examinations of a factory in Japan Japan [18]	7 years	2,037 men 35–55 years	170 incident cases of T2D Consumption of one or more servings per week of diet soda associated with increased diabetes risk after multivariable adjustment (HR: 1.70; 95% CI: 1.13–2.55; P = 0.013) compared to non-consumers of diet soda.
O’Connor L, et al. 2015 The EPIC-Norfolk Study UK [19]	10.8 years	24,653 adults 40–79 years	847 incident cases of T2D One serving per day (336 g) of artificially sweetened beverages was associated to development of T2D after multivariable adjustment (HR: 1.22; 95% CI: 1.11–1.33; P<0.001). After a second adjustment considering adiposity (BMI and waist circumference) the association did not remained significant (HR: 1.06; 95% CI: 0.93–1.20; P = 0.124)

T2D: type 2 diabetes, BMI: body mass index, RR: relative risk, CI: confidence interval, HR: hazard ratio, OR: odds ratio.

**Table 2:** Crude and adjusted associations between the consumption of artificially sweetened beverages and the development of metabolic diseases in observational prospective studies.

Cohort	Pathology	Follow-up	n	Crude risk	Multivariable adjustment	Adiposity adjustment
NHS I [7]	T2D	24 years	74,749	1.59 (1.47–1.71)	1.35 (1.24–1.47)	1.01 (0.93–1.10)
NHS II [8]	T2D	8 years	91,249	1.21 (0.97–1.50)	----	----
Framingham Heart Study [11]	MS	4 years	6,039	1.42 (1.10–1.84)	1.53 (1.10–2.15)	----
ARIC [12]	MS	9 years	9,514	1.20 (1.11–1.29)	1.34 (1.24–1.44)	----
BWHS [14]	T2D	4 years	43,960	1.06 (0.83–1.36)	----	----
MESA [15]	MS	7 years	5,011	1.31 (1.07–1.60)	1.36 (1.11–1.66)	1.17 (0.96–1.44)
MESA [15]	T2D	7 years	5,011	1.63 (1.24–2.13)	1.67 (1.27–2.20)	1.38 (1.04–1.82)
HPFS [7]	T2D	22 years	39,059	1.87 (1.65–2.12)	1.32 (1.15–1.51)	1.06 (0.93–1.22)
HPFS– 2 [9]	T2D	20 years	40,389	1.91 (1.72–2.11)	1.40 (1.26–1.56)	1.09 (0.98–1.21)
CARDIA^a^ [16]	MS	20 years	3,728	0.81 (0.69–0.95)	----	----
SAHS [13]	OB	7–8 years	3,682	2.03 (1.36–3.03)	----	----
EPIC [10]	T2D	16 years	340,234	1.93 (1.47–2.54)	1.88 (1.44–2.45)	1.13 (0.85–1.52)
EPIC-France [17]	T2D	14 years	66,118	3.50 (2.49–4.93)	2.21 (1.56–3.14)	1.68 (1.19–2.39)
EPIC-Norfolk [19]	T2D	10.8 years	24,653	1.70 (1.35–2.14)	1.67 (1.33–2.11)	1.17 (0.93–1.48)
Employee Factory Japan [18]	T2D	7 years	2,037	1.99 (1.33–2.98)	1.82 (1.22–2.71)	1.70 (1.13–2.55)

NHS: Nurses’ Health Study, ARIC: the Atherosclerosis Risk in Communities study, BWHS: the Blacks Women’s Health Study, MESA: the Multi-Ethnic Study of Atherosclerosis, HPFS: the Health Professionals Follow-Up Study, CARDIA: the Coronary Artery Risk Development in Young Adults study, SAHS: the San Antonio Heart Study, EPIC: the European Prospective Investigation into Cancer and Nutrition study, T2D: type 2 diabetes, MS: metabolic syndrome, OB: obesity, n: individuals included in the studies. Associations between the highest range of artificially sweetened beverages consumption and the incidence of the specific metabolic disease studied, expressed in relative risks, odds ratios or hazard ratios with 95% confidence intervals (95% CI).

^a^The CARDIA study evaluates the risk of the non-consumers of diet beverages to develop metabolic syndrome compared to the consumers.

### Clinical trials

Twenty-eight clinical trials studying different effects of NNS were identified. Of these studies, 10 found significant effects on some or all the studied variables. All of these studies have analyzed glucose and most of them have measured insulin concentrations, 11 quantified GLP-1 concentrations. However, only one study has measured insulin sensitivity and pancreatic response, and another single study has evaluated the changes in the intestinal microbiome. The majority of the clinical trials have evaluated the effects of aspartame (14 trials), followed by sucralose (11 studies), and saccharin, acesulfame-K, and stevia (5 studies for saccharin, 5 for acesulfame-K, and 4 for stevia). Most of these studies have performed an acute single exposure to the NNS (n = 20) and the remaining (n = 8) have evaluated a longer exposure that varies between seven days to 18 weeks. Thirteen studies included individuals with diabetes.

The studies by Pepino [20] and Suez [21] demonstrate a deleterious effect increasing glucose concentrations after an acute and a 7-day exposure to sucralose and saccharin, respectively. Pepino, also reported a decrease in insulin sensitivity along with increased insulin and C-peptide concentrations. Remarkably, this study included subjects with a high degree of obesity (average BMI 42 kg/m2). In the study of Suez after a seven-day period of saccharin ingestion, in four of seven subjects glucose concentrations showed a significant increment. Subsequently, a feces transplant from some of the individuals with the glucose increase after saccharin exposure to mice was performed. After the transplant, glucose concentrations also increased in these mice, suggesting that NNS consumption modify intestinal microbiome in detriment of glucose tolerance. The microbiome showed a significant imbalance with an increase in the *Bacteroides* genus and Clostridiales order [21].

GLP-1 concentrations, measured in eleven studies, have been shown to be decreased in one report after aspartame ingestion [22] and increased in two studies after sucralose + acesulfame-K and sucralose exposure [23, 24]. Concentrations of appetite-regulating hormones, including cholecystokinin, ghrelin, and peptide YY, have only been studied in three studies. In none of them changes in the concentrations of these variables were found. In addition, no change in the subjective appetite ratings or on the quantity of food consumed after NNS exposure has been found.

On Table 3 the description and results of these studies are shown. As a reference, one 12 oz diet-coke contains approximately 140 mg of aspartame and acesulfame K mix, one 12 oz diet-Dr. Pepper can contains approximately 65 and 22 mg of sucralose and acesulfame, respectively, and one 12-oz Coca-Cola Life can contains 27 mg of stevia. Some of the NNS available as individual packets include Sweet and Low, containing 34 mg of saccharin, and Splenda containing 12 mg of sucralose. On Table 4 a summary of the studies indicating the methodology used, studied variables, and the NNS evaluated are presented.

**Table 3:** Clinical trials evaluating the effect of non-nutritive sweeteners consumption on glucose metabolism and appetite regulating hormones.

No.	Author and year	Population	Methodology	Variables	Results
1	Nehrling JK, et al. 1985 [25]	62 subjects with diabetes (31 insulin-dependent and 31 non-insulin-dependent) aged 18–65 years	Randomized, double-blind study 29 participants consumed 2.7 g of aspartame per day in capsules and 33 participants consumed placebo capsules (corn starch) during 18 weeks Fasting and 2-hour after breakfast samples collected in weeks 0, 9, 17, 18	Glucose HbA1c	No changes in plasma glucose or HbA1c levels during the treatment.
2	Okuno G, et al. 1986 [26]	First study: 7 healthy controls and 22 untreated subjects with diabetes aged 18–64 years Second study: 9 subjects with diabetes aged 38–81 years (5 treated with insulin)	First study: crossover design, 2 visits, consumption of 100 g glucose or 500 mg aspartame on fasting Second study: daily consumption of 125 mg aspartame over 2 weeks, OGTT (50 g glucose) before and after intervention	Glucose Insulin Glucagon Triglycerides Total cholesterol HDL-cholesterol	Small but significant decrease in blood glucose 2 h and 3 h after aspartame administration compared to glucose in first study (p<0.05) No other changes were observed in both studies after the consumption of aspartame
3	Horwitz DL, et al. 1988 [27]	12 normal subjects and 10 subjects with non-insulin-dependent diabetes aged 18–65 years	Crossover study 3 visits: consumption of a flavored beverage unsweetened or with 135 mg saccharin or 400 mg aspartame Samples collected over 3 h after consuming the test beverage	Glucose Insulin Glucagon	No significant effects of sweeteners at any time point in glucose, insulin or glucagon In normal subjects, higher mean AUC insulin levels after aspartame compared with saccharin or unsweetened beverage (p<0.05)
4	Cooper PL, et al. 1988 [28]	17 subjects with non-insulin-dependent diabetes, aged 62.2±14.0 years, and BMI 26.0±3.0 kg/m^2^	Crossover study Daily intake of 28 g sucrose or 30 g starch + saccharin during 6 weeks Samples collected over 3 hours at the beginning of the study and at the end of each intervention period	Glucose Insulin Triglycerides	No changes on glucose, insulin or triglycerides were found with the saccharin ingestion
5	Colagiuri S, et al. 1989 [29]	9 subjects with non-insulin-dependent diabetes, aged 66±5 years, and BMI 26.4±2.1 kg/m^2^	Crossover study Daily intake of 45 g sucrose or 162 mg aspartame during 6 weeks Samples collected on fasting at the beginning of the study and at the end of each intervention period	Glucose HbA1c Weight Total cholesterol HDL-cholesterol Triglycerides	Aspartame ingestion did not generate changes on any of the variables measured
6	Rodin J 1990 [30]	12 overweight and 12 normal-weight subjects, aged 22–50 years	Crossover study 4 visits: 500 ml water or water + 50 g glucose or 50 g fructose or 250 mg aspartame Samples collected over 48 minutes; later, subjects consumed a lunch until they felt satiated	Glucose Insulin Glucagon Free fatty acids Caloric intake	Aspartame consumption had not effects on glucose, insulin, glucagon and free fatty acids concentrations The aspartame load did not stimulate food intake beyond the consumption of water (control)
7	Härtel B, et al. 1993 [31]	14 healthy subjects aged 19–52 years with normal glucose tolerance	Crossover study 6 visits: 330 ml water only or water + 33 g sucrose or 165 mg aspartame or 165 mg acesulfame-K or 800 mg cyclamate or 75 mg saccharin Blood Samples collected over 120 minutes	Glucose Insulin	Lower insulin levels after the NNS ingestion compared to sucrose (p<0.001) Lower glucose levels in some times after aspartame or saccharin ingestion compared to water (p<0.05), this changes were not physiologically meaningful
8	Mezitis NH, et al. 1996 [32]	13 subjects with T1D and 13 subjects with T2D (HbA1c <10%)	Crossover study 2 visits: administration of one capsule with 1000 mg sucralose or placebo (cellulose), followed by a standardized 360-Kcal liquid breakfast Blood samples obtained during 4 hours	Glucose C-peptide	Sucralose ingestion had no effects on glucose and C-peptide concentrations compared to placebo Hypoglycemia occurred in each of three T1D participants with the sucralose ingestion; however, sucralose was not considered the cause
9	Melanson KJ, et al. 1999 [33]	10 healthy non-smorkers men, aged 19–31 years, BMI 23.4±1.9 kg/m^2^	Crossover study 3 visits: consumption of simple carbohydrate or high-fat or aspartame-containing drinks Later, subjects consumed food *ad libitum*	Glucose Caloric intake	In 40% of the participants, blood glucose declined after aspartame ingestion, while in 20% increased and in 40% remained stable No statistically significant differences between groups on caloric intake
10	Grotz VL, et al. 2003 [34]	128 subjects with T2D, aged 31–70 years, and HbA1c levels ≤10%	2 randomized assigned groups: daily consumption of two capsules with sucralose (667 mg per day) or two capsules of placebo (cellulose) during 13 weeks	Glucose C-peptide HbA1c	No effects were found on glucose, C-peptide or changes in HbA1c after sucralose consumption
11	Hall WL, et al. 2003 [22]	6 subjects aged 24–31 years and BMI <25 kg/m^2^	Crossover study 3 visits: ingestion of capsules with 400 mg aspartame or 176 mg aspartic acid + 224 mg phenylalanine or 400 mg corn flour as control Samples collected over 120 minutes VAS to measure subjective appetite ratings	Glucose Insulin GLP-1 GIP CCK Gastric emptying Desire to eat Hunger Fullness	Lower plasma GLP-1 concentrations after aspartame and amino acids ingestion (p<0.05). Aspartame consumption had not effects on the other variables
12	Gregersen S, et al. 2004 [35]	12 subjects with T2D, BMI 25–32 kg/m^2^, and HbA1c levels <10%	Crossover study 2 visits: 412 kcal breakfast consumption + supplement (1 g stevioside or 1 g maize starch as control) Samples collected over 240 minutes	Glucose Insulin GLP-1 GIP Glucagon Insulinogenic index	Stevioside reduced the glycemic response in 18±5% (p = 0.013) Insulinogenic index increased by approximately 40% after stevioside consumption (p<0.001) No other statistically significant effects were found on insulin, glucagon, GLP-1 and GIP
13	Barriocanal LA, et al. 2008 [36]	76 subjects divided in 3 groups: 30 with T2D, 16 with T1D, and 30 healthy subjects Each group was subdivided to receive the active treatment or placebo	Randomized assignment to consume 250 mg steviol glycosides or placebo Participants were followed-up for 3 months	Glucose Insulin HbA1c	Steviol glycosides did not generate changes on any of the studied variables
14	Maki KC, et al. 2008 [37]	122 subjects with diabetes aged 33–75 years	Randomized double-blind study 60 subjects consumed 1000 mg rebaudioside A capsules and 62 subjects consumed placebo capsules (cellulose) during 16 weeks Subjects were asked to maintain a stable diet during the study	HbA1c Glucose Insulin C-peptide Body weight Blood pressure Triglycerides Total cholesterol HDL-cholesterol LDL-cholesterol	The consumption of rebaudioside A over 16 weeks did not shown effects in any variable
15	Ma J, et al. 2009 [38]	7 healthy subjects with BMI 21.6±1.2 kg/m^2^, age 24±2 years, non-smokers, and alcohol consumption <20 g per day	Crossover study 4 visits: intragastric infusion of 50 g sucrose, 80 mg sucralose, 800 mg sucralose or 500 ml saline in 3 minutes Samples were obtained during 240 minutes	Glucose Insulin GLP-1 GIP Gastric emptying	Sucralose did not showed effects at any dose on glucose, insulin, GLP-1, GIP, and gastric emptying compared to saline
16	Anton SD, et al. 2010 [39]	Subjects aged 18–49 years and non-smokers. 19 subjects with normal weight and 12 with obesity (waist circumference at least 90 cm for females and 100 cm for males)	Crossover study 3 visits: consumption of tea sweetened with sucrose or stevia or aspartame (quantity not specified) previous to the consumption of a buffet *ad libitum* VAS to measure subjective appetite ratings	Glucose Insulin Insulinogenic index Hunger Satiety Fullness Organoleptic characteristics	Lower plasma glucose and insulin concentrations with stevia consumption compared to sucrose (p<0.01 for glucose and p<0.05 for insulin) Greater insulinogenic index with aspartame consumption at 60 minutes (p<0.05) Energy intake did not increase with NNS consumption and no effects were found on appetite parameters
17	Ma J, et al. 2010 [40]	10 healthy subjects, with BMI 23.4±0.8 kg/m^2^, and age 27±2 years	Crossover study 2 visits: intraduodenal infusion of sucralose (960 mg) in saline compared to only saline infusion during 150 minutes	Glucose GLP-1	No effects on glucose intestinal absorption or GLP-1 secretion were observed with sucralose consumption
18	Ford HE, et al. 2011 [41]	8 healthy subjects aged 22–27 years, with BMI 18.8 kg/m^2^, and non-smokers	Crossover study 3 visits: ingestion of 50 ml water or sucralose or maltodextrin + sucralose After solutions ingestion, modified-sham-feeding protocol was executed (stimulation of oral cavity sweet-taste receptors) Blood samples were obtained during 2 hours	Glucose Insulin GLP-1 PYY Food intake Hunger Desire to eat Cephalic response	Sucralose did not stimulate cephalic response and had no effects on glucose, insulin, GLP-1 and PYY concentrations Sucralose did not showed differences in appetite subjective ratings or food intake
19	Brown AW, et al. 2011 [23]	8 female volunteers with BMI 22.16±1.71 kg/m^2^, aged 21.75±2.25 years, non-smokers, without diabetes or alcohol consumption	Crossover study 4 visits: 355 ml water or water + 50 g sucrose or 6 g granular sucralose or 50 g sucrose and 6 g granular sucralose Breakfast (500 kcal) after 60 minutes and blood samples over the next 2 hours VAS to measure appetite	Glucose Insulin Glucagon Triglycerides Ghrelin Hunger Gastrointestinal comfort General well-being	No significant differences were observed in any of the variables with the consumption of sucralose compared to water
20	Steinert RE, et al. 2011 [42]	12 healthy subjects aged 23.3±0.7 years, BMI 23.0±0.5 kg/m^2^, non-smokers and without chronic diseases	Crossover study 6 visits: intragastic infusion (over 2 min) of 250 ml water or water + 50 g glucose or 25 g fructose or 169 mg aspartame or 220 mg acesulfame-K or 62 mg sucralose Blood samples obtained during 2 hours VAS to measure appetite	Glucose Insulin GLP-1 PYY Ghrelin Hunger Satiety Fullness	None of the NNS had effects on biochemical variables compared to water Lower appetite subjective ratings with NNS compared to glucose and fructose; however, the differences were not statistically significant
21	Maersk M, et al. 2012 [43]	24 subjects aged 20–50 years with obesity (BMI 28–36 kg/m^2^) Individuals with diabetes or pregnancy were excluded	Crossover study 4 visits: 500 ml sucrose-sweetened regular soda, 500 ml semi-skimmed milk, 500 ml aspartame-sweetened diet soda or 500 ml bottled still water. *Ad libitum* food consumption from a buffet after 4 hours VAS to measure appetite	Glucose Insulin Ghrelin GLP-1 GIP Hunger Satiety Fullness Prospective desire to eat ThirstEnergy intake	Aspartame-containing beverage did not showed effects on any of the variables
22	Wu T, et al. 2012 [44]	10 healthy subjects aged 28.8±4.0 years, and BMI 25.5±1.5 kg/m^2^	Crossover study 4 visits: ingestion of 40 g glucose, 40 g tagatose/ isomalt mixture, 40 g 3-O-methylglucose, or 60 mg sucralose Samples collected over 240 minutes	Glucose GLP-1 GIP Insulin Gastric emptying	Sucralose consumption did not present effects on glucose, insulin, GLP-1 and GIP concentrations Gastric emptying was lower after the ingestion of tagatose/ isomalt mixture and 3-O-methylglucose compared to sucralose (p = 0.033 and p = 0.012, respectively)
23	Brown R, et al. 2012 [45]	Subjects aged 12–25 years divided in 3 groups: 9 with T1D, 10 with T2D, and 25 healthy control participants All T2D were overweight or obese	Crossover study 3-h OGTT with 75 g 2 visits: at minute -10 subjects drank 240 ml of diet soda with sucralose and acesulfame-K or 240 ml of carbonated water	Glucose C-peptide GLP-1 GIP PYY	GLP-1 AUC 43% higher with the ingestion of diet soda in T1D subjects (p = 0.02) GLP-1 AUC 34% higher with the ingestion of diet soda in healthy subjects (p = 0.029) No differences on glucose, C-peptide, GIP, and PYY
24	Olalde-Mendoza L, et al. [46] 2013	80 subjects with T2D aged 49.3±9.06 years, BMI 30.5±4.30 kg/m^2^, and less than 10 years of diabetes evolution	Randomized study: 40 subjects consumed 200 ml of diet soda containing 40 mg/100 g of an aspartame and acesulfame-K mix. The other 40 subjects consumed 200 mL of regular soda Samples collected at 0, 10, 15 and 30 minutes after the ingestion of beverages	Capillary glucose	No effects of diet soda on capillary glucose levels
25	Pepino Y, et al. 2013 [20]	17 subjects with BMI 42.3±1.6 kg/m^2^ with low previous NNS consumption (less than one can of diet beverage or one spoonful of NNS per week)	Crossover study 5 hours OGTT with 75 g 2 visits: at minute -10 subjects drank 60 ml of only water or 60 ml of water + 48 mg of sucralose	Glucose Insulin GLP-1 GIP Glucagon C-peptide Insulin sensitivity β-Cell function Insulin clearance	Higher concentrations in some times for glucose, insulin and C-peptide (p<0.004) Insulin clearance decrease in 7±4% (p = 0.04) Insulin sensitivity decrease 23±20% (p = 0.01) No differences in GLP-1, GIP, glucagon and the pancreatic response
26	Suez J, et al. 2014 [21]	7 subjects aged 28–36 years followed for 7 days Not normally consumers of NNS or NNS-containing foods (criteria not specified) No specification of other characteristics of participants	Consumption of 100% ADI of commercial saccharin (5 mg per kg of body weight) during 6 days Daily OGTT Gut microbiota was analyzed on day 1 and 7 A placebo-controlled group was not included	Glucose Changes in gut microbiota	4 of the 7 subjects presented higher glucose concentrations in days 5–7 (p<0.001) Fecal transplantation of NNS-responding subjects to germ-free mice increased the glucose concentrations in mice (p<0.05)
27	Bryant CE, et al. 2014 [47]	10 subjects with BMI 21.8±21.8 kg/m^2^ No comment of other characteristics of participants	Crossover study 4 visits: ingestion of 45 g glucose, 45 g glucose + 150 mg aspartame, 45 g glucose + 20 mg saccharin, 45 g glucose + 85 mg acesulfame-K VAS to measure appetite	Glucose Hunger Fullness	NNS did not showed effects on glucose, hunger or fullness. Acesulfame-K glucose AUC 17.4% higher compared with only glucose ingestion; however, it was no statistically significant
28	Temizkan S, et al. 2015 [24]	8 newly diagnosed T2D subjects without pharmacological treatment, aged 51.5±9.2 years and 8 apparently healthy subjects aged 45.0±4.1 years	Crossover study 3 visits: 2 hour OGTT 75 g At minute -15 subjects drank 200 ml water or water + 24 mg sucralose or 72 mg aspartame	Glucose Insulin GLP-1 C-peptide	Lower glucose AUC (p = 0.002) and higher GLP-1 AUC (p = 0.04) with sucralose compared to water in healthy participants No effects of NNS on insulin and C-peptide No differences in any of the variables in T2D subjects

BMI: body mass index, HbA1c: glycated hemoglobin, HDL: high density lipoproteins, LDL: low density lipoproteins, T1D: type 1 diabetes, T2D: type 2 diabetes, VAS: visual analogue scales, GLP-1: glucagon like peptide type 1, GIP: glucose-dependent insulinotropic peptide, CCK: cholecystokinin, PYY: tyrosine tyrosine peptide, NNS: non-nutritive sweeteners, OGTT: oral glucose tolerance test, AUC: area under the curve.

**Table 4:** Summary of the studied variables, non-nutritive sweetener used, study methodology and findings of the clinical trials evaluated in Table 3.

	Study number (according to Table 3)																											
	1	2	3	4	5	6	7	8	9	0	11	12	13	14	15	16	17	18	19	20	21	22	23	24	25	26	27	28
**Glucose**																												
**Insulin**																												
**GLP-1**																												
**GIP**																												
**HbA1c**																												
**C-peptide**																												
**Glucagon**																												
**PYY**																												
**Ghrelin**																												
**CCK**																												
**Triglycerides**																												
**VAS to measure appetite**																												
**Caloric intake**																												
**Insulin sensitivity**																												
**β-Cell function**																												
**Insulin clearance**																												
**Insulinogenic index**																												
**Gut microbiota**																												
**Gastric emptying**																												
Saccharin																												
Aspartame																												
Acesulfame-K																												
Sucralose																												
Stevia																												
Short-term exposition																												
Crossover design																												
Had found effects*																												

GLP-1: glucagon like peptide type 1, GIP: glucose-dependent insulinotropic peptide, HbA1c: glycated hemoglobin, PYY: tyrosine tyrosine peptide, CCK: cholecystokinin, VAS: visual analogue scales

*This refers to studies that have found significant changes in some or all the studied variables without signifying positive or negative effects. For review of these effects, please refer to Table 3.

### Meta-analysis

Two meta-analyses have been published to evaluate the association between the consumption of NNS-containing beverages and the development of type 2 diabetes to clarify if this relation is clearly linked to the consumption of these products or related to other lifestyle factors. Both meta-analyses evaluated the association between NNS consumption, without specifying of stratifying for the specific NNS ingested. While both studies excluded cohorts including individuals with a known diagnosis of diabetes, the article by Grenwood only included four studies. This may be due to the selection criteria that specify that only studies including individuals “from a generally healthy population” were considered [48]. In contrast, the study by Imamura evaluated ten studies estimating the risk of type 2 diabetes associated to consumption of NNS-containing beverages [49]. None of the studies disclosed significant competing interests.

In the first meta-analysis that included 4 observational prospective studies, the pooled estimated relative risk (RR) was 1.13 (95% CI: 1.02–1.25; P = 0.02) for the consumption of 330 ml per day of artificially sweetened beverages and the development of type 2 diabetes. There was high heterogeneity between studies, and the positive association was less consistent for this type of beverages compared to the sugar-sweetened drinks [48].

In the second meta-analysis with 10 studies included, the crude RR was 1.48 (95% CI: 1.35–1.62; P<0.05). However, after adjustment for BMI and the calibration for information and publication bias, the association was no longer statistically significant (RR: 1.22; 95% IC: 0.98–1.52; P = 0.07) [49].

## Discussion

The aim of this systematic review is to evaluate the scientific available evidence regarding the association between NNS consumption and metabolic diseases as well as the effects of NNS on glucose metabolism and appetite regulating hormones. The results indicate that the association between NNS intake and the development of metabolic diseases, mainly type 2 diabetes, is not clear. A common identified confounding factor in the observational prospective studies is adiposity. In addition, it is unknown if the NNS are associated with deleterious effects on glucose metabolism or appetite regulation. Based on the available evidence, an effect of NNS on glucose metabolism cannot be established. The study of appetite and its regulation is complex, the evidence presented concerning this issue is scarce and an effect of NNS in appetite cannot be demonstrated either. The studies found are varied regarding the NNS studied; therefore, a class effect cannot be determined and no solid conclusions regarding a specific NNS can be stated.

A possible explanation for the associations found in some of the observational studies among NNS consumption and the development of metabolic diseases might be that these cohorts included participants prone to develop these outcomes, for example with family members with diabetes or with a predisposition for weight gain, that are likely to consume these products. For example, people with higher BMI, already at risk to develop diabetes, consume NNS-containing beverages as a strategy to minimize calorie intake.

An additional limitation of observational studies is that the majority has evaluated the consumption of NNS containing-beverages and the non-consuming population may actually have consumed these substances from other non-acknowledged products. Finally, the evidence level of observational studies cannot establish causality.

Most of the clinical trials included have small sample sizes and the majority does not provide a justification for these calculations. Many of the clinical trials are crossover studies and a main limitation of this design is the residual effect between treatments. In most cases there is no information regarding the washout period. Another variable that needs to be considered is the amount of NNS used and the exposition type, for example acute or long-term exposition. Moreover, there is no uniformity in the exposition time between studies evaluating a long-term exposure. Finally, a number of confounding variables are not mentioned or adjusted in these trials, including BMI, previous NNS intake, and presence of metabolic alterations such as glucose intolerance or diabetes, among others. These drawbacks may confuse the results presented.

We can conclude that some clinical trials have found effects of NNS on glucose metabolism. However the results are contradictory and there is no possible comparison between the trials due to the heterogeneity in the population included, NNS studied, placebo use, exposure time, outcomes evaluated, among many other. For example, after sucralose consumption, one study reported higher concentrations of glucose, however, another study report lower concentrations and nine studies did not observed changes in glucose. In addition, two studies found that sucralose increase GLP-1 levels compared to water, an effect that other six studies could not confirm. One study found that sucralose decreases insulin sensitivity and insulin clearance in morbid obese population, nevertheless, this is the only one trial that has evaluated these outcomes.

The consumption of aspartame showed lower concentrations of glucose in two of fourteen studies, one compared to water and the other one to glucose. One study observed lower concentrations of insulin after aspartame *vs*. sucrose and another study found higher concentrations of insulin after aspartame *vs*. water. Finally, one trial reported that aspartame decreases GLP-1 concentrations compared to placebo.

For stevia, one trial observed lower glucose and insulin concentrations compared to sucrose, and another study found lower glucose concentrations and an increment in the insulinogenic index compared to placebo.

One trial reflected an important impact of saccharin consumption for seven days promoting glucose intolerance in four of seven subjects studied; this trial suggest that this effect is caused by altering the gut microbiome performing a fecal transplantation to mice showing a similar increase in glucose levels. In contrast, one study showed lower glucose and insulin concentrations after saccharin ingestion compared to water or sucrose, respectively.

The findings of the two meta-analyses should be interpreted cautiously. In the first report few studies were included, without considering other variables that may be involved in the development of diabetes, and in the second the association between NNS-containing beverages and development of diabetes was lost after the adjustment for body mass index, indicating that adiposity may be influencing the findings.

Randomized clinical trials testing each of the NNS, including a homogeneous group of participants, without metabolic conditions that may confound the results, including an adequate sample size, with an appropriate control group, during an appropriate exposure time, and considering adjustment or control for significant variables such as adiposity are needed. In addition, the mechanisms involved in the glucose metabolism changes after a long-term exposition to NNS should explored in human studies.

Based on the scientific evidence presented, the consumption of NNS is not encouraged, but they could be considered a useful tool in the nutritional treatment of certain metabolic diseases as sugar substitutes as long as the quantity consumed is within the acceptable daily intake (ADI) and without compensation by ingesting other energy-rich foods. Lastly, health professionals should not promote the consumption of sweet tasting foods regardless its source.

## Supporting Information

S1 File PRISMA checklist. (DOC)
